# Silencing H19 regulated proliferation, invasion, and autophagy in the placenta by targeting miR‐18a‐5p

**DOI:** 10.1002/jcb.28172

**Published:** 2018-12-09

**Authors:** Lei Zhang, Xinru Deng, Xian Shi, Xiaojing Dong

**Affiliations:** ^1^ Department of Obstetrics and Gynecology The Second Affiliated Hospital, Chongqing Medical University Chongqing China

**Keywords:** autophagy, fetal growth restriction (FGR), H19, invasion, miR‐18a‐5p, proliferation

## Abstract

Fetal growth restriction (FGR) is a serious pregnancy complication associated with increased perinatal mortality and morbidity. It may lead to neurodevelopmental impairment and adulthood onset disorders. Recently, long noncoding RNAs (lncRNAs) were found to be associated with the pathogenesis of FGR. Here we report that the lncRNAH19 is significantly decreased in placentae from pregnancies with FGR. Downregulation of H19 leads to reduced proliferation and invasion of extravillous trophoblast cells. This is identified with reduced trophoblast invasion, which has been discovered in FGR. Autophagy is exaggerated in FGR. Downregulation of H19 promotes autophagy via the PI3K/AKT/mTOR and MAPK/ERK/mTOR pathways of extravillous trophoblast cells in FGR. We also found that the expression level of microRNAs miR‐18a‐5p was negatively correlated with that of H19. H19 can act as an endogenous sponge by directly binding to miR‐18a‐5p, which targets IRF2. The expression of miR‐18a‐5p was upregulated, but IRF2 expression was downregulated after the H19 knockdown. In conclusion, our study revealed that H19 downexpressed could inhibit proliferation and invasion, and promote autophagy by targeting miR‐18a‐5pin HTR8 and JEG3 cells. We propose that aberrant regulation of H19/miR‐18a‐5p‐mediated regulatory pathway may contribute to the molecular mechanism of FGR. We indicated that H19 may be a potential predictive, diagnostic, and therapeutic modality for FGR.

## 1 INTRODUCTION

Fetal growth restriction (FGR) is defined as the fetal weight below the tenth percentile for gestational age or the birth weight less than 2500 g after 37 weeks of gestation. FGR increases the incidence of fetal distress and neonatal asphyxia/hypoglycemia^1^ and is the second leading cause of perinatal death. FGR is also a risk factor of adult hypertension, neurocognitive delay, and metabolic disorders.^2^ Furthermore, there is yet a lack of eﬀective therapeutic strategies. Therefore, exploration of the underlying pathogenesis is urgently needed.

Long noncoding RNAs (lncRNAs) are a class of nonprotein‐coding RNA transcripts with over 200 nucleotides,^3^ which are involved in the regulation of many cellular processes, such as tumor growth, apoptosis, proliferation, invasion, and autophagy.^4^ The lncRNA H19 is transcribed from a maternally expressed imprinted gene that locates on the chromosome 11p15.5,^5^ and an upregulation of H19 has been detected in certain cancers (breast,^6^ stomach, and bladder^7^, ^8^). MiRNAs, which act as oncogenes or tumor suppressors, were found to be abnormally expressed in diverse cancers by degrading or repressing translations of the messenger RNA (mRNA) targets.^9^ It is known that lncRNAs sometimes function by acting as precursors of miRNAs.^10^, ^11^ lncRNAs can directly bind with particular DNA or RNA strands to participate in the transcriptional and posttranscriptional gene regulation^12^ and can act as “miRNA sponge” to inhibit the activity of miRNA, thus affecting the expression of miRNA target genes.^13^ The interplay of miRNAs and lncRNAs was reported to exert an important regulatory role in tumors.^14^, ^15^ Previous data have revealed that H19 may play a part in FGR.^16^ Therefore, we hypothesized that there might be a relationship between lncRNA‐H19 and FGR progression.

The placenta is the crucial organ for embryo‐fetal development and plays a critical role in the genesis of FGR.^17^ The invasion of trophoblast‐derived cells into maternal uterine tissue is a specific and constant feature of human placentation.^18^ Extravillous trophoblast cells migrate and invade into the uterine wall, leading to remodeling of the maternal vasculature.^16^ Inappropriate invasion may lead to pregnancy diseases, such as FGR and pre‐eclampsia.

Autophagy is a conserved intracellular self‐degrading by lysosome,^19^ which is the main degradation response to the stresses such as hypoxia and starvation.^20^ Autophagy is prosurvival or prodeath, depending on the types of stressors and cells.^21^ The autophagy process is mainly modulated by mTOR‐dependent or mTOR‐independent pathways, and the mTOR‐dependent pathway is the most common route to activate autophagy.^22^ It is reported that autophagy is increased in FGR. The expression of autophagy markers, including LC3‐II and Beclin‐1, is upregulated in placenta from patients with FGR compared with normal pregnant women.^23^ Therefore the changes in cell viability, invasion, and autophagy are key to the pathophysiology of FGR.

In this study, the correlation between the H19 expression level and FGR and the underlying mechanisms were explored. The preliminary data demonstrated that an enhancement of autophagy due to the H19/miR‐18a‐5p pathway was involved in the pathogenesis of FGR.

We found that H19 was significantly decreased in human placenta tissues with FGR compared with normal pregnancy. We demonstrate that downexpression of H19 may decrease trophoblast cell HTR8 and choriocarcinoma cell JEG3 proliferation and invasion. It also promotes autophagy by targeting miR‐18a‐5p. We hypothesized that dysregulation of this newly identified H19/miR‐18a‐5p‐mediated regulatory pathway may contribute to the underlying mechanism of FGR.

## 2 MATERIALS AND METHODS

### 2.1 Tissue specimens

A total of 20 pairs of placenta tissues were sampled from the Second Affiliated Hospital of Chongqing Medical University. The tissue specimens were aliquoted and then stored at −80°C (for protein extraction) or in RNA storage fluid (for RNA extraction). The use of human tissues was ethically approved by the Institutional Review Board.

### 2.2 Cell culture and transfection

Human trophoblast cells HTR8 and choriocarcinoma cells JEG3 (Beijing Beina Chuanglian Biotechnology Institute, Beijing, China) were cultured in RPMI‐1640 medium (Corning Inc., New York state, NY) containing 12% fetal bovine serum (FBS; PAN, Bavaria, Germany) at 37°C and 5% CO_2_ concentration. Small interfering RNAs (siRNAs) for H19 and negative control, and miR‐18a‐5p mimics and inhibitor were synthesized from GenePharma Co Ltd (Shanghai, China). The siRNAs, miR‐18a‐5p mimics, and inhibitors were transfected into HTR8 and JEG3 cells using a transfection kit from GenePharma Co Ltd. The sequences were H19 siRNA, 1977, 5′‐CCCGUCCCUUCUGAAUUUATTUAAAUUCAGAAGGGACGGGTT‐3′; miR‐18a‐5p mimics, 5′‐UAAGGUGCAUCUAGUGCAGAUAGAUCUGCACUAGAUGCACCUUAUU‐3′; miR‐18a‐5p inhibitor, 5′‐CUAUCUGCACUAGAUGCACCUUA‐3′.

### 2.3 Cellular invasion

A transwell invasion assay was performed using Boyden's chamber. Cells were transfected and subsequently dissociated, and the transfected cells were resuspended in 200 µL serum‐free MEM medium and placed in the upper chamber with an 8‐µm pore, 6.5‐mm polycarbonate filter (Corning Inc). The cells were planted in the upper chamber of Matrigel‐coated (Corning Inc) with serum‐free MEM medium and incubated for 24 hours. The insert was placed in a well with medium containing 12% FBS. The cells that did not migrate through the pores were removed by a wet cotton swab after 24 hours. The lower membrane cells were fixed in 4% paraformaldehyde for 30 minutes and stained with 0.5% crystal violet (Beyotime, Jiangsu, China). A total of five randomly selected fields were observed under a microscope, and the cell number was counted (magnification, ×200).

### 2.4 5‐Ethynyl‐2′‐deoxyuridine‐based proliferation assay

Cell proliferation was measured by the EdU DNA Cell Proliferation kit (C10310; Guangzhou RiboBio Co, Ltd, China).

### 2.5 Cell viability assay

Cell viability was determined by a WST‐8 assay (Hanbio Biotechnology Co Ltd, Shanghai, China). Cells were plated in a 96‐well plate, and siRNA was transfected after 24 hours. The absorbance was measured with a multifunctional microplate reader (Thermo Fisher Scientific) at 450 nm at 24, 48, and 72 hours.

### 2.6 RNA isolation and real‐time polymerase chain reaction

Total RNA was extracted from cells and tissues using the High‐purity Total RNA Rapid Extraction kit (RP1201; BioTeke Corporation, Beijing, China; CA). cDNA was synthesized using the All‐in‐One First‐Strand cDNA Synthesis Kit (GeneCopoeia, Guangzhou, China) or the miRNA First Strand cDNA Synthesis kit (GeneCopoeia). The primers for H19, β‐actin were 5′‐CCACGGAGTCGGCACACTATG‐3′ and 5′‐GAGCTGGGTAGCACCATTTCTTTC‐3′ for H19, and 5′‐ACTAAGGTGCATCTAGTGCAGATAG‐3′ for miR‐18a‐5p. The real‐time polymerase chain reaction (PCR) was performed using the All‐in‐One qPCR Mix Kit (GeneCopoeia) or the microRNAs qPCR Kit (SYBR Green method) kit (GeneCopoeia). Gβ‐actin or U6 was the reference (Bio‐Rad, CA).

### 2.7 Western blot analysis

Total protein was extracted using the cell lysis buffer, and the concentration was measured with the BCA assay (Beyotime). Proteins (40 μg) were separated with SDS‐PAGE and then transferred to a PVDF membrane (Millipore, Bedford, MA). The membrane was blocked with 8% skim milk (Difco Laboratoies, Detroit, MI) for 1 hour and incubated with primary rabbit antibody overnight at 4°C, including monoclonal anti‐PI3 Kinase Class III (D9A5; #4263; Cell Signaling Technology, MA), monoclonal anti‐AKT (1:1000; #4691; Cell Signaling Technology), monoclonal anti‐p‐AKT (1:1000; #4060; Cell Signaling Technology), monoclonal anti‐MAPK (1:1000; #8690; Cell Signaling Technology), monoclonal anti‐ERK1/2(1:1000; #4695; Cell Signaling Technology), monoclonal anti‐p‐ERK1/2(1:1000; #4730; Cell Signaling Technology), monoclonal anti‐mTOR (7C10; 1:1000; #2983; Cell Signaling Technology), monoclonal anti‐p‐mTOR(Ser2448; 1:1000; #5536; Cell Signaling Technology), monoclonal rabbit anti‐ULK1 antibody (D8H5; 1:1000; #8054; Cell Signaling Technology), monoclonal anti‐Beclin1 (1:1000; #3495; Cell Signaling Technology), monoclonal anti‐P62 (1:1000; #5114; Cell Signaling Technology), monoclonal anti‐LC3 LC3A/B (D3U4C; 1:1000; #12741; Cell Signaling Technology), or polyclonal anti‐β‐actin antibody (1:1000; #4970; Cell Signaling Technology). The membranes were then washed with TBST (three times, 10 minutes/time), incubated with the HRP‐conjugated secondary antibody at room temperature for 2 hours, and then washed with TBST (three times, 10 minutes/time). Proteins were visualized with the ECL system (Beyotime) using the ChemiDoc XRS system (Bio‐Rad).

### 2.8 Dual‐luciferase reporter gene assay

For the H19 3′‐untranslated region (3′‐UTR) luciferase reporter assay, wild‐type or mutant reporter constructs (termed wt or mut; Gene Pharma Co Ltd, Shanghai, China), and miR‐18a‐5p control or miR‐18‐5p mimics, were cotransfected into HTR8 and JEG3 cells using Lipofectamine 2000 (Invitrogen, CA). After 48 hours, luciferase activity was measured using the Dual Luciferase Assay System, with Renilla activity as the reference.

### 2.9 Statistical analysis

All statistical analyses were performed using the software GraphPad6.0 (GraphPad Inc, San Diego, CA) and SPSS (version 24.0; Chicago, IL). The Student *t* test or analysis of variance was used to determine statistical significance. The statistical significance was set at *P* < 0.05.

## 3 RESULTS

### 3.1 lncRNA H19 expression level was downregulated in FGR placenta tissues

**Figure 1:**
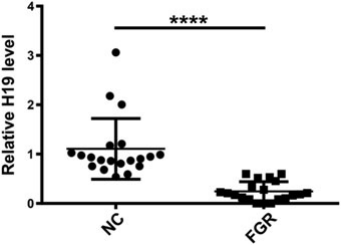
The expression level of H19 in placenta tissues are significantly decreased in the FGR placentae. Expression of lncRNA H19 in placenta tissues from patients with FGR and full‐term pregnancy women were measured by qRT‐PCR. Data were presented as the mean ± SD of 20 independent experiments. ^****^ *P* < 0.0001. FGR, fetal growth restriction; lncRNA, long noncoding RNA; SD, standard deviation

The placenta tissues of normal and FGR pregnancy was sampled. The PCR demonstrated a lower level of H19 in FGR placenta in comparison with the control placenta (Figure 1; *P* < 0.0001). The mRNA level of β‐actin in each sample was also quantified as an internal standard and used to normalize the levels of H19 from the same sample.

### 3.2 Downregulation of H19 suppressed cell proliferation and invasion

**Figure 2:**
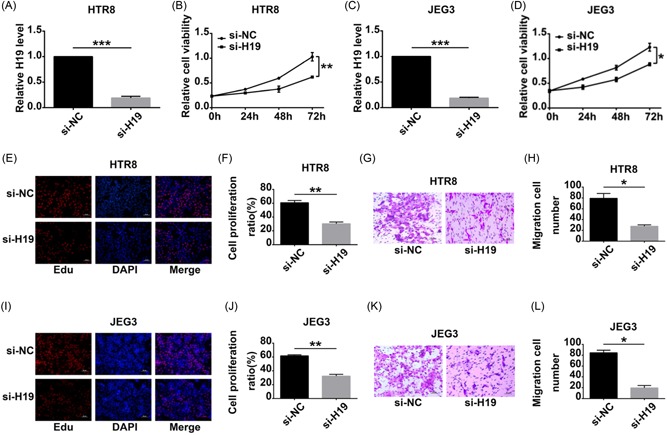
H19‐regulated proliferation and invasion of HTR8 and JEG3 cells (A) and (C). The effect of siRNAs on silencing H19 in HTR8 and JEG3 B and D. CCK‐8 was performed to determined cell proliferation. The data show that cell proliferation was decreased in H19‐silenced HTR8 and JEG3 cells (E,F,I,J). EdU‐based proliferation assay was performed 48 hours after RNA interference. At the proliferation stage, the nuclei were dyed red with EdU. All nuclei were dyed blue with DAPI. Red and blue were merged into magenta to show the proportion of nuclei with proliferation. Proliferation of H19‐silenced cells was clearly inhibited (G,H,K,L). Cellular invasion was tested by transwell assays. The number of cells passed through the membrane was counted and compared in the diagrams. The invaded cells was decreased in si‐H19 when compared with si‐NC. *P < 0.05; ^**^ *P* < 0.01; ^***^ *P* < 0.001. CCK‐8, cell counting kit‐8; EdU, 5‐ethynyl‐2′‐deoxyuridine; NC, negative control; siRNA, small interfering RNA

Human trophoblast cells HTR8 and choriocarcinoma cells JEG3 were used for silencing trials. PCR showed that the expression level of H19 was decreased in HTR8 and JEG3 cells after siRNA transfection (Figure 2A; *P* = 0.0006; Figure 2C; *P* = 0.0001). Cell Counting Kit‐8 (CCK‐8) and 5‐ethynyl‐2′‐deoxyuridine assays indicated that the percentage of proliferative cells was decreased in H19‐silenced cells (Figure 2B; *P* = 0.0090; Figure 2E and 2F; *P* = 0.0108; Figure 2D; *P* = 0.0474; Figure 2I and 2J; *P* = 0.0040). Silencing H19 inhibited the cellular invasion (Figure 2G and 2H; *P* = 0.0161; Figure 2K and 2L; *P* = 0.0048).

### 3.3 H19 regulated autophagy via the PI3K/AKT‐mTOR and MAPK/ERK‐mTOR pathways

**Figure 3:**
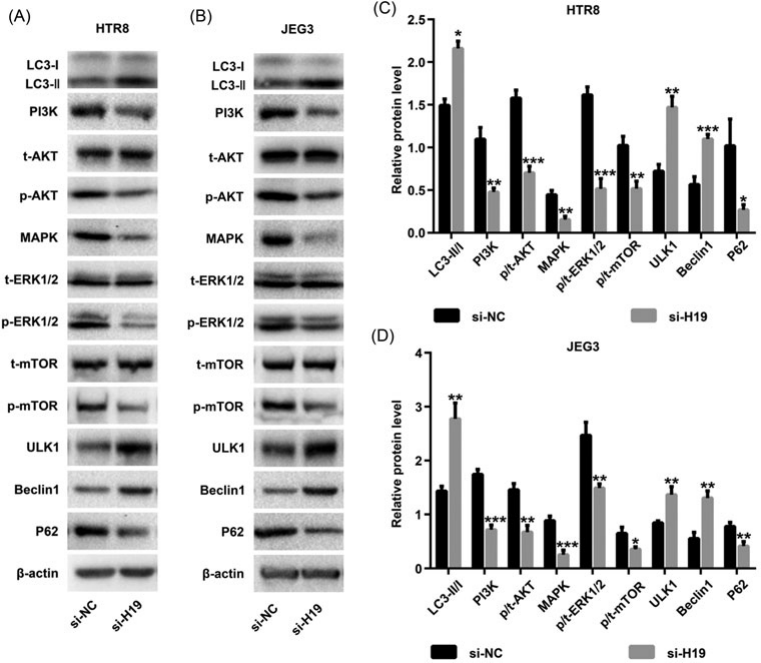
lncRNA H19 inhibits autophagy in HTR8 and JEG3 cells. Expressions of key kinases involved in the PI3K/AKT‐mTOR and MAPK/ERK‐mTOR pathways in HTR8 cells (A,C) and JEG3 cells (B,D) were assessed by Western blot analysis. Data were presented as the mean ± SD of three independent experiments. *P < 0.05; ^**^ *P* < 0.01; ^***^ *P* < 0.001. lncRNA, long noncoding RNA

The autophagy biomarker LC3 was determined by Western blot. Knockdown of H19 resulted in an increase in the ratio of LC3‐II/LC3‐I, indicating enhancement of autophagy (Figure 3A‐D).

To explore the mechanisms of H19 in regulating autophagy, t‐AKT, p‐AKT, PI3K, MAPK, t‐ERK1/2, p‐ERK1/2, t‐mTOR, p‐mTOR, ULK1, Beclin1, and P62 were detected. After silencing H19, the level of p‐AKT, PI3K, MAPK, p‐ERK1/2, p‐mTOR, and P62 was decreased, and the level of ULK1 and Beclin1 was increased; however, the level of t‐AKT, t‐ERK1/2 and t‐mTOR was not altered.

### 3.4 H19 regulated IRF2 through miR‐18a‐5p

**Figure 4:**
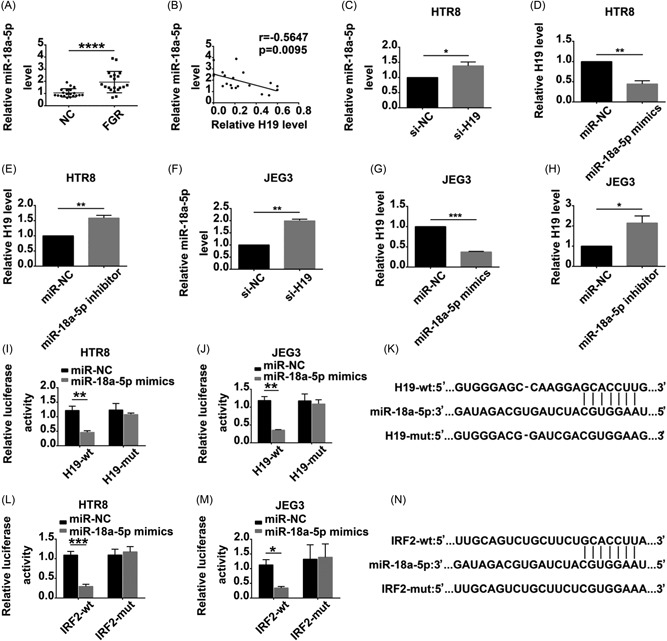
lncRNA H19 regulates IRF2 through binding with miR‐18a‐5p in HTR8 and JEG3 cells. A, The expression of miR‐18a‐5p was significantly increased in the FGR placentae. B, The correlation between H19 and miR‐18a‐5p was proved by Spearman's correlation method. C,F, miR‐18a‐5p was upregulated in H19‐silenced HTR8 and JEG3. D,G, The expression of H19 was decreased after transfection with miR‐18a‐5p mimics. E,H, The expression of H19 was increased after transfection with miR‐18a‐5p inhibitor. I,J,L,M, Dual‐luciferase assay was also performed to confirm the interaction between H19 and miR‐18a‐5p, miR18a‐5p and IRF2. K,N, The binding sites were obtained between two types of H19 and IRF2 (wild‐type and mutant‐type) with miR‐18a‐5p. *P < 0.05; ^**^ *P* < 0.01; ^***^ *P* < 0.001; ^****^ *P* < 0.0001. FGR, fetal growth restriction; lncRNA, long noncoding RNA

lncRNAs can function as competing endogenous RNAs (ceRNAs) to sponge miRNA. Analyses utilizing the online target sequence prediction database (http://starbase.sysu.edu.cn/) suggested that miR‐18a‐5p may have potential binding sites to H19, and therefore the expression level of miR‐18a‐5p mRNA in placenta tissues was determined by qRT‐PCR. The level of miR‐18a‐5p mRNA in FGR placenta tissues was increased, but that of H19 was decreased, compared with control (Figure 4A). The miR‐18a‐5p level negatively correlated with the H19 level (Figure 4B). miR‐18a‐5p expression was promoted after H19 downregulation (Figure 4C and 4F). H19 expression was inhibited after transfection with miR‐18a‐5p mimics (Figure 4D and 4G) and was increased after transfected with the miR‐18a‐5p inhibitor (Figure 4E and 4H).

To verify whether miR‐18a‐5p was targeted and directly bound to lncRNA‐H19, a luciferase reporter vector containing the predicted wild‐type or mutated miR‐18a‐5p binding sites in H19 was constructed (Figure 4K). As shown in HTR8 (Figure 4I) and JEG3 (Figure 4J), the overexpression of miR‐18a‐5p remarkably reduced the luciferase intensity in the H19‐3′‐UTR wild‐type. In addition, the luciferase reporter system revealed that miR‐18a‐5p overexpression suppressed the luciferase activity of pGL3‐IRF2‐3′‐UTR‐WT (Figure 4N) in HTR8 (Figure 4L) and JEG3 (Figure 4M). These data indicated that miR‐18a‐5p suppressed H19 expression by directly binding to its 3′‐UTR and that IRF2 was a target of miR‐18a‐5p.

### 3.5 lncRNA H19/miR‐18a‐5p impacted on cell proliferation and invasion

**Figure 5:**
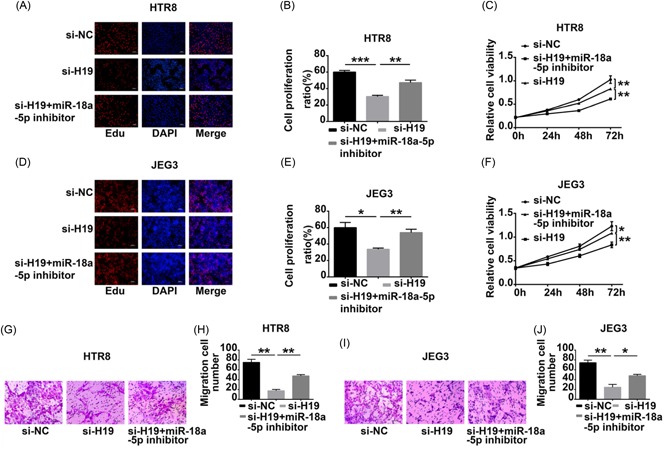
lncRNA H19/miR‐18a‐5p regulate cell proliferation and invasion. A‐F, EdU‐based proliferation assay and CCK‐8 were used to assess cell proliferation. Data shown the proliferation were reversed after cotransfected with si‐H19 and miR‐18a‐5p inhibitor compared with transfection of si‐H19. G‐J, Transwell invasion assay were used to assess cell invasion. Data shown the invasion were reversed after cotransfected with si‐H19 and miR‐18a‐5p inhibitor compared with transfection of si‐H19. *P < 0.05; ^**^ *P* < 0.01; ^***^ *P* < 0.001. CCK‐8, cell counting kit‐8; EdU, 5‐ethynyl‐2′‐deoxyuridine; lncRNA, long noncoding RNA

**Figure 6:**
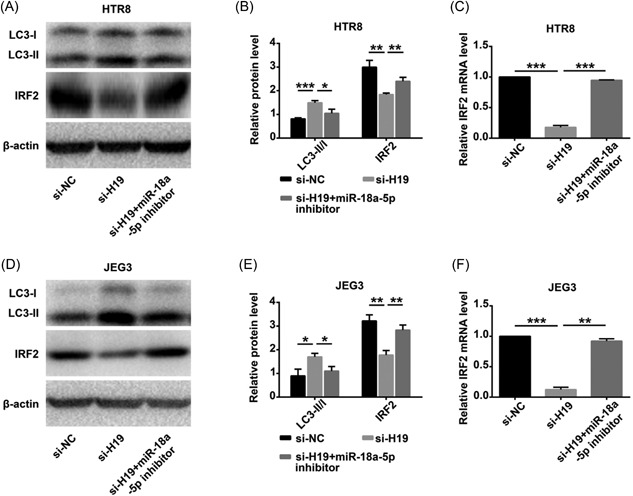
lncRNA H19/miR‐18a‐5p regulate IRF2 and autophagy. A,B,D,E, In downexpressed H19 group, the expression of IRF2 and the ratio of LC3‐II/LC3‐I were significantly increased in protein level. Whereas the expression of IRF2 and the ratio of LC3‐II/LC3‐I were reversed in the cotransfecting group. C,F, The expression of IRF2 in mRNA level were measured by qRT‐PCR. *P < 0.05; ^**^ *P* < 0.01; ^***^ *P* < 0.001. lncRNA, long noncoding RNA; mRNA, messenger RNA

The effects of lncRNA‐H19/miR‐18a‐5p on cell proliferation and invasion were explored. The cells were transfected with si‐H19 or were cotransfected with si‐H19 and miR‐18a‐5p inhibitors. Downexpression of H19 decreased the ability of proliferation and invasion in HTR8 and JEG3 cells. This effect was reversed when cotransfecting si‐H19 and miR‐18a‐5p inhibitor in HTR8 (Figure 5A‐C, 5G, and 5H) and JEG3 (Figure 5D‐F, 5I, and 5J). Cotransfection also reversed the enhancement of autophagy attributable to knockdown of H19 (Figure 6A and 6B) and JEG3 (Figure 6D and 6E). miR‐18a‐5p inhibitor increased the level of IRF2 (Figure 6C and 6F). However, the modulating effects of H19 on IRF2 were diminished after cotransfection with the miR‐18a‐5p inhibitor.

## 4 DISCUSSION

In this study, we observed a decreased expression of H19 in the placenta of FGR. We also found that silencing H19 inhibited proliferation and invasion and promoted autophagy of extravillous trophoblast cells by targeting miR‐18a‐5p. PI3K/AKT/mTOR and MAPK/ERK/mTOR pathways were regulated by H19. Our results indicated that H19 was a potential treatment target for FGR.

Previous studies have reported that H19 was downregulated in papillary thyroid carcinoma compared with adjacent paracancerous tissues. Inhibition of H19 expression can promote the proliferation, migration, and tumor growth.^24^ The expression level of H19 was notably higher in tongue squamous cell carcinoma. H19 promotes tongue squamous cell carcinoma invasion through β‐catenin/GSK3β/EMT signaling via association with EZH2.^25^ Accumulating evidence highlighted that aberrant expression of H19 was closely related to embryogenesis, fetal growth, and tumorigenesis.^26^ Proper development of human placenta is essential for the maintenance of normal pregnancy. In this study, the biological function of H19 in HTR8 and JEG3 cells was explored. Downregulation of H19 reduced the cellular proliferation and invasion. Proliferation of trophoblast cells was involved in the placental development,^27^ and the invasion of trophoblast cells into the uterine spiral artery was the determining process for the formation of uteroplacental circulation.^28^ Inadequate invasion and low viability of trophoblast cells may be a reason of FGR. These results indicated that the downexpression of H19 was associated with FGR. Recently, a growing number of research studies have suggested that the interaction between lncRNA H19 and miRNAs played an effective role in the gene regulation and was involved in various biological processes. lncRNAs can act as ceRNA sponges for miRNAs to regulate the downstream genes to regulate physiological and pathological processes.^29^ In human placental trophoblast cell, H19 can produce miR‐675 that targets Nodal Modulator 1, and trophoblast cell proliferation was decreased after downexpression of H19.^30^ In ovarian cancer cell lines, SKOV‐3 and OVCAR3 cells, H19 could directly bind to miR‐370‐3p and effectively act as its ceRNA. H19 overexpression or a miR‐370‐3p knockdown promoted TGF‐β‐induced EMT.^31^ In glioma cells U251 and U87, H19 promotes proliferation and invasion by downregulating miR‐152.^32^ H19 gene may participate in placenta development by a posttranscriptional mechanism.^30^ In our study, the data showed that the expression of miR‐18a‐5p was negatively correlated with the level of H19 in FGR placenta tissues. Downexpression of H19 increased the activity of miR‐18a‐5p, followed by a decrease of the downstream target IRF2. Luciferase activity assay indicated that H19 and miR‐18a‐5p directly regulated each other. H19 served as a molecular sponge for miR‐18a‐5p to regulate its target IRF2. The suppression in proliferation and invasion in HTR8 and JEG3 cells due to H19 downexpression was reversed when transfected with the miR‐18a‐5p inhibitor. Moreover, downregulation of H19 suppressed cell proliferation and invasion by negatively regulating miR‐18a‐5p expression. This study provided important clues to understand the interaction between H19 and miR‐18a‐5p in FGR.

Autophagy has been generally reported to play a key role in development, aging, neurological disorders, liver and muscle disorders, infection, immunity, and cancer.^33^, ^34^, ^35^ The Basal level of autophagy is responsible for the maintaining of cellular homeostasis; however, excessive autophagy may induce autophagic cell death.^36^ lncRNA is known to regulate autophagy through diverse mechanisms. In this regard, lncRNA is able to target some autophagy protein related microRNAs to regulate autophagy.^37^ Therefore, the effect of lncRNA‐H19 on autophagy in trophoblast cells may affect the progression of FGR. Previous data demonstrated that autophagy was enhanced in FGR placenta.^20^, ^23^ In the placenta of FGR, autophagic vacuoles containing lysosomal enzymes and semi‐digested cell organelles were more prominent, indicating that placental autophagy might be associated with pathophysiology of FGR.^19^ Beclin‐1 is a major component for autophagic initiation and participates in the formation of autophagosomes.^38^ P62 is the best‐known autophagic substrate, which binds to ubiquitinated proteins and is consumed during autophagosomal degradation.^39^, ^40^ ULK1 is the downstream of mTOR and upstream of Beclin1.^41^ Autophagosome formation is involved in the conversion of the soluble form of LC3‐I to the autophagic vesicle‐associated form LC3‐II.^42^ Thus, the increased ratio of LC3B‐II/LC3B‐I and expression of Beclin‐1, as well as decreased p62 expression after H19 downexpressed, proved that silencing H19 promoted autophagy. There are several signal pathways underlying the regulation of cell autophagy including the PI3K/Akt/mTOR, Bcl2/beclin1, MAPK/Erk1/2, and AMPK signaling pathways.^43^, ^44^ The PI3K, Akt, MAPK, and Erk pathways may regulate autophagy.^45^, ^46^ The PI3K/AKT and mTOR pathways are essential signaling cascades that are involved in cell proliferation.^47^ The trophoblast invasion is decreased as the phosphorylation of mTOR is prevented.^48^ The ERK/MAPK pathway is an evolutionarily conserved signaling pathway that responds to signals from cell surface receptors to promote cell growth, proliferation, and survival.^49^ In our study, the enhancement of autophagy was observed after silencing H19. H19 downexpression promoted autophagy via the PI3K/AKT/mTOR and MAPK/ERK/mTOR pathways. Cotransfection with miR‐18a‐5p inhibitor reverses the enhancement of autophagy, which is attributable to knockdown of H19. The more validated the autophagy is in association with FGR may unveil novel strategies for disease therapy.

In conclusion, H19 was downregulated in FGR placenta. H19 regulated cellular proliferation, invasion, and autophagy through targeting miR‐18a‐5p. H19 served as a molecular sponge for miR‐18a‐5p to regulate IRF2. An aberrant H19/miR‐18a‐5p‐mediated regulatory pathway may participate in the occurrence of FGR. These data indicated that H19 in trophoblast cells may be a therapeutic target for FGR.

## Supporting information

Supporting information

## References

[1] American College of Obstetricians and Gynecologists . ACOG practice bulletin no. 134: fetal growth restriction. Obstet Gynecol. 2013;121(5):1122‐1133. 23635765 10.1097/01.AOG.0000429658.85846.f9

[2] Wang KCW , Zhang L , McMillen IC , et al. Fetal growth restriction and the programming of heart growth and cardiac insulin‐like growth factor 2 expression in the lamb. J Physiol. 2011;589(Pt 19):4709‐4722. 21807611 10.1113/jphysiol.2011.211185 PMC3213418

[3] Lu Y , Li Y , Chai X , et al. Long noncoding RNA HULC promotes cell proliferation by regulating PI3K/AKT signaling pathway in chronic myeloid leukemia. Gene. 2017;607:41‐46. 28069548 10.1016/j.gene.2017.01.004

[4] Chen S , Wu DD , Sang XB , et al. The lncRNA HULC functions as an oncogene by targeting ATG7 and ITGB1 in epithelial ovarian carcinoma. Cell Death Dis. 2017;8(10):e3118. 29022892 10.1038/cddis.2017.486 PMC5682654

[5] Wu T , Qu L , He G , et al. Regulation of laryngeal squamous cell cancer progression by the lncRNA H19/miR‐148a‐3p/DNMT1 axis. Oncotarget. 2016;7(10):11553‐11566. 26872375 10.18632/oncotarget.7270 PMC4905493

[6] Berteaux N , Lottin S , Monté D , et al. H19 mRNA‐like noncoding RNA promotes breast cancer cell proliferation through positive control by E2F1. J Biol Chem. 2005;280(33):29625‐29636. 15985428 10.1074/jbc.M504033200

[7] Li H , Yu B , Li J , et al. Overexpression of lncRNA H19 enhances carcinogenesis and metastasis of gastric cancer. Oncotarget. 2014;5(8):2318‐2329. 24810858 10.18632/oncotarget.1913 PMC4039165

[8] Luo M , Li Z , Wang W , Zeng Y , Liu Z , Qiu J . Long noncoding RNA H19 increases bladder cancer metastasis by associating with EZH2 and inhibiting E‐cadherin expression. Cancer Lett. 2013;333(2):213‐221. 23354591 10.1016/j.canlet.2013.01.033

[9] Wu Z , Han Y , Li Y , et al. MiR‐218‐5p inhibits the stem cell properties and invasive ability of the A2B5^+^CD133^−^ subgroup of human glioma stem cells. Oncol Rep. 2016;35(2):869‐877. 26572167 10.3892/or.2015.4418

[10] Ebert MS , Sharp PA . Roles for microRNAs in conferring robustness to biological processes. Cell. 2012;149(3):515‐524. 22541426 10.1016/j.cell.2012.04.005 PMC3351105

[11] Huntzinger E , Izaurralde E . Gene silencing by microRNAs: contributions of translational repression and mRNA decay. Nat Rev Genet. 2011;12(2):99‐110. 21245828 10.1038/nrg2936

[12] Karapetyan AR , Buiting C , Kuiper RA , Coolen MW . Regulatory roles for long ncRNA and mRNA. Cancers. 2013;5(2):462‐490. 24216986 10.3390/cancers5020462 PMC3730338

[13] Gutschner T , Diederichs S . The hallmarks of cancer: a long noncoding RNA point of view. RNA Biol. 2012;9(6):703‐719. 22664915 10.4161/rna.20481 PMC3495743

[14] Liu Q , Huang J , Zhou N , et al. LncRNA loc285194 is a p53‐regulated tumor suppressor. Nucleic Acids Res. 2013;41(9):4976‐4987. 23558749 10.1093/nar/gkt182 PMC3643595

[15] Jalali S , Bhartiya D , Lalwani MK , Sivasubbu S , Scaria V . Systematic transcriptome‐wide analysis of lncRNA‐miRNA interactions. PLoS One. 2013;8(2):e53823. 23405074 10.1371/journal.pone.0053823 PMC3566149

[16] Zuckerwise L , Li J , Lu L , et al. H19 long noncoding RNA alters trophoblast cell migration and invasion by regulating TβR3 in placentae with fetal growth restriction. Oncotarget. 2016;7(25):38398‐38407. 27223264 10.18632/oncotarget.9534 PMC5122399

[17] Chiofalo B , Laganà AS , Vaiarelli A , et al. Do miRNAs play a role in fetal growth restriction? A fresh look to a busy corner. BioMed Res Int. 2017;2017:6073167‐6073168. 28466013 10.1155/2017/6073167 PMC5390605

[18] Doridot L , Miralles F , Barbaux S , Vaiman D . Trophoblasts, invasion, and microRNA. Front Genet. 2013;4:248. 24312123 10.3389/fgene.2013.00248 PMC3836020

[19] Zhang QX , Na Q , Song W . Altered expression of mTOR and autophagy in term normal human placentas. Rom J Morphol Embryol. 2017;58(2):517‐526. 28730238

[20] Curtis S , Jones CJP , Garrod A , Hulme CH , Heazell AEP . Identification of autophagic vacuoles and regulators of autophagy in villous trophoblast from normal term pregnancies and in fetal growth restriction. J Matern Fetal Neonatal Med. 2013;26(4):339‐346. 23039021 10.3109/14767058.2012.733764

[21] Conte A , Paladino S , Bianco G , et al. High mobility group A1 protein modulates autophagy in cancer cells. Cell Death Differ. 2017;24(11):1948‐1962. 28777374 10.1038/cdd.2017.117 PMC5635219

[22] Ravikumar B , Futter M , Jahreiss L , et al. Mammalian macroautophagy at a glance. J Cell Sci. 2009;122(Pt 11):1707‐1711. 19461070 10.1242/jcs.031773 PMC2684830

[23] Hung TH , Chen SF , Lo LM , Li MJ , Yeh YL , Hsieh TT . Increased autophagy in placentas of intrauterine growth‐restricted pregnancies. PLoS One. 2012;7(7):e40957. 22815878 10.1371/journal.pone.0040957 PMC3397998

[24] Lan X , Sun W , Dong W , et al. Downregulation of long noncoding RNA H19 contributes to the proliferation and migration of papillary thyroid carcinoma. Gene. 2018;646:98‐105. 29287713 10.1016/j.gene.2017.12.051

[25] Zhang DM , Lin ZY , Yang ZH , et al. IncRNA H19 promotes tongue squamous cell carcinoma progression through β‐catenin/GSK3β/EMT signaling via association with EZH2. Am J Transl Res. 2017;9(7):3474‐3486. 28804564 PMC5527262

[26] Ratajczak MZ . Igf2‐H19, an imprinted tandem gene, is an important regulator of embryonic development, a guardian of the proliferation of adult pluripotent stem cells, a regulator of longevity, and a 'passkey' to cancerogenesis. Folia Histochem Cytobiol. 2012;50(2):171‐179. 22763974 10.5603/fhc.2012.0026

[27] Huppertz B . The anatomy of the normal placenta. J Clin Pathol. 2008;61(12):1296‐1302. 18755720 10.1136/jcp.2008.055277

[28] Regnault TRH , de Vrijer B , Galan HL , et al. The relationship between transplacental O2 diffusion and placental expression of PlGF, VEGF and their receptors in a placental insufficiency model of fetal growth restriction. J Physiol. 2003;550(Pt 2):641‐656. 12740423 10.1113/jphysiol.2003.039511 PMC2343042

[29] Deng G , Sui G . Noncoding RNA in oncogenesis: a new era of identifying key players. Int J Mol Sci. 2013;14(9):18319‐18349. 24013378 10.3390/ijms140918319 PMC3794782

[30] Gao W , Liu M , Yang Y , et al. The imprinted H19 gene regulates human placental trophoblast cell proliferation via encoding miR‐675 that targets Nodal Modulator 1 (NOMO1). RNA Biol. 2012;9(7):1002‐1010. 22832245 10.4161/rna.20807

[31] Li J , Huang Y , Deng X , et al. Long noncoding RNA H19 promotes transforming growth factor‐β‐induced epithelial‐mesenchymal transition by acting as a competing endogenous RNA of miR‐370‐3p in ovarian cancer cells. Onco Targets Ther. 2018;11:427‐440. 29403287 10.2147/OTT.S149908 PMC5783024

[32] Chen L , Wang Y , He J , Zhang C , Chen J , Shi D . Long noncoding RNA H19 promotes proliferation and invasion in human glioma cells by downregulating miR‐152. Oncol Res. 2018 10.3727/096504018X15178768577951 PMC7844716 29422115

[33] Hale AN , Ledbetter DJ , Gawriluk TR , Rucker EB, III . Autophagy: regulation and role in development. Autophagy. 2013;9(7):951‐972. 24121596 10.4161/auto.24273 PMC3722331

[34] Levine B , Kroemer G . Autophagy in the pathogenesis of the disease. Cell. 2008;132(1):27‐42. 18191218 10.1016/j.cell.2007.12.018 PMC2696814

[35] Sermersheim MA , Park KH , Gumpper K , et al. MicroRNA regulation of autophagy in cardiovascular disease. Front Biosci. 2017;22:48‐65. 10.2741/4471 PMC5349319 27814601

[36] Yonekawa T , Thorburn A . Autophagy and cell death. Essays Biochem. 2013;55:105‐117. 24070475 10.1042/bse0550105 PMC3894632

[37] Wang J , Cao B , Han D , Sun M , Feng J . Long noncoding RNA H19 induces cerebral ischemia‐reperfusion injury via activation of autophagy. Aging Dis. 2017;8(1):71‐84. 28203482 10.14336/AD.2016.0530 PMC5287389

[38] Jin S , Tian S , Chen Y , et al. USP19 modulates autophagy and antiviral immune responses by deubiquitinating Beclin‐1. EMBO J. 2016;35(8):866‐880. 26988033 10.15252/embj.201593596 PMC4972138

[39] Lee Y , Weihl CC . Regulation of SQSTM1/p62 via UBA domain ubiquitination and its role in disease. Autophagy. 2017;13(9):1615‐1616. 28812433 10.1080/15548627.2017.1339845 PMC5612413

[40] Au AK , Aneja RK , Bayır H , et al. Autophagy biomarkers Beclin 1 and p62 are increased in cerebrospinal fluid after traumatic brain injury. Neurocrit Care. 2017;26(3):348‐355. 28000126 10.1007/s12028-016-0351-x

[41] Lin H , Wang T , Ruan Y , et al. Rapamycin supplementation may ameliorate erectile function in rats with streptozotocin‐induced type 1 diabetes by inducing autophagy and inhibiting apoptosis, endothelial dysfunction, and corporal fibrosis. J Sex Med. 2018;15(9):1246‐1259. 30224017 10.1016/j.jsxm.2018.07.013

[42] Nishida K , Kyoi S , Yamaguchi O , Sadoshima J , Otsu K . The role of autophagy in the heart. Cell Death Differ. 2009;16(1):31‐38. 19008922 10.1038/cdd.2008.163

[43] Hou X , Hu Z , Xu H , et al. Advanced glycation endproducts trigger autophagy in cadiomyocyte via RAGE/PI3K/AKT/mTOR pathway. Cardiovasc Diabetol. 2014;13:78. 24725502 10.1186/1475-2840-13-78 PMC3998738

[44] Kim YC , Guan KL . mTOR: a pharmacologic target for autophagy regulation. J Clin Invest. 2015;125(1):25‐32. 25654547 10.1172/JCI73939 PMC4382265

[45] Campbell GR , Bruckman RS , Herns SD , Joshi S , Durden DL , Spector SA . Induction of autophagy by PI3K/mTOR and PI3K/mTOR/BRD4 inhibitors suppresses HIV‐1 replication. J Biol Chem. 2018;293(16):5808‐5820. 29475942 10.1074/jbc.RA118.002353 PMC5912471

[46] Xu X , Zhi T , Chao H , et al. ERK1/2/mTOR/Stat3 pathway‐mediated autophagy alleviates traumatic brain injury‐induced acute lung injury. Biochim Biophys Acta. 2018;1864(5 Pt A):1663‐1674. 10.1016/j.bbadis.2018.02.011 29466698

[47] Shrivastava S , Kulkarni P , Thummuri D , et al. Piperlongumine, an alkaloid causes inhibition of PI3 K/Akt/mTOR signaling axis to induce caspase‐dependent apoptosis in human triple‐negative breast cancer cells. Apoptosis. 2014;19(7):1148‐1164. 24729100 10.1007/s10495-014-0991-2

[48] Knuth A , Liu L , Nielsen H , Merril D , Torry DS , Arroyo JA . Placenta growth factor induces invasion and activates p70 during rapamycin treatment in trophoblast cells. Am J Reprod Immunol. 2015;73(4):330‐340. 25271148 10.1111/aji.12327

[49] Yoon S , Seger R . The extracellular signal‐regulated kinase: multiple substrates regulate diverse cellular functions. Growth Factors. 2006;24(1):21‐44. 16393692 10.1080/02699050500284218

