# Supplementary figures and images for: Silencing H19 regulated proliferation, invasion, and autophagy in the placenta by targeting miR‐18a‐5p
Source: J Cell Biochem. 2018 Dec 9;120(6):9006–15. doi: 10.1002/jcb.28172 (PMC6587755; doi:10.1002/jcb.28172)

Supplementary Figure


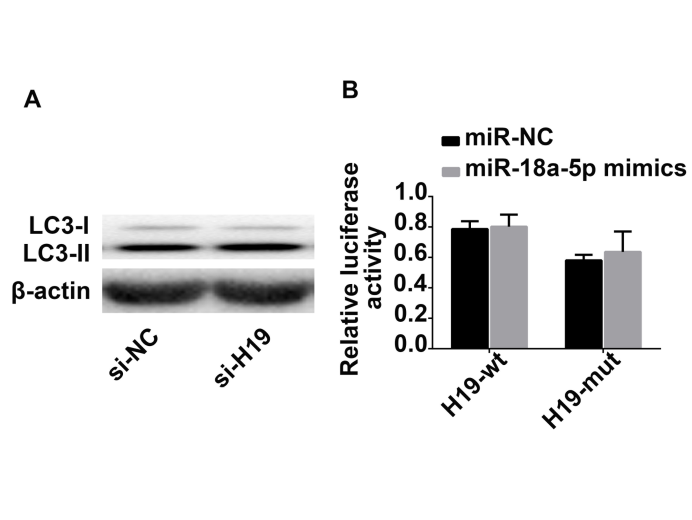

Supplement: Supplementary file 1 — Supporting information [file JCB-120-9006-s001.docx]
